# PCSK9 inhibition and cholesterol homeostasis in insulin producing β-cells

**DOI:** 10.1186/s12944-022-01751-6

**Published:** 2022-12-16

**Authors:** Günter Päth, Nikolaos Perakakis, Christos S. Mantzoros, Jochen Seufert

**Affiliations:** 1 Division of Endocrinology and Diabetology, Department of Medicine II, Medical Center – University of Freiburg, Faculty of Medicine, University of Freiburg, Germany, Hugstetter Str. 55, Freiburg, Germany; 2 Division of Metabolic and Vascular Medicine, Department of Internal Medicine III, University Hospital Carl Gustav Carus, Technische Universität Dresden, Dresden, Germany; 3 Division of Endocrinology, Diabetes and Metabolism, Department of Internal Medicine, Beth Israel Deaconess Medical Center, Harvard Medical School, Boston, MA USA; 4 Section of Endocrinology, VA Boston Healthcare System, MA Jamaica Plain, USA

**Keywords:** PCSK9 inhibitors, Glucose homeostasis, Insulin secretion, Loss of function gene variants, Knockout models, Pancreatic β-cells

## Abstract

Low-density lipoprotein cholesterol (LDL-C) plays a central role in the pathology of atherosclerotic cardiovascular disease. For decades, the gold standard for LDL-C lowering have been statins, although these drugs carry a moderate risk for the development of new-onset diabetes. The inhibitors of proprotein convertase subtilisin/kexin type 9 (PCSK9) have emerged in the last years as potential alternatives to statins due to their high efficiency and safety without indications for a diabetes risk so far. Both approaches finally eliminate LDL-C from bloodstream by upregulation of LDL receptor surface expression. Due to their low antioxidant capacity, insulin producing pancreatic β-cells are sensitive to increased lipid oxidation and related generation of reactive oxygen species. Thus, PCSK9 inhibition has been argued to promote diabetes like statins. Potentially, the remaining patients at risk will be identified in the future. Otherwise, there is increasing evidence that loss of circulating PCSK9 does not worsen glycaemia since it is compensated by local PCSK9 expression in β-cells and other islet cells. This review explores the situation in β-cells. We evaluated the relevant biology of PCSK9 and the effects of its functional loss in rodent knockout models, carriers of LDL-lowering gene variants and PCSK9 inhibitor-treated patients.

## PCSK9 inhibitors improve dyslipidaemia without diabetes risk

Circulating low-density lipoprotein cholesterol (LDL-C) has long been recognised for its central role in the development and progression of atherosclerosis, which is the pathological basis of cardiovascular disease (CVD). According to a 2021 World Health Organisation report, CVD is the leading cause of morbidity and mortality worldwide [1], and is particularly associated with worse outcomes in patients with diabetes [2]. Lowering of LDL-C is therefore an important clinical goal, which is commonly addressed with statins as the first choice therapy.

Statins inhibit cholesterol synthesis by blocking the rate-limiting 3-hydroxy-3-methyl-glutaryl-coenzyme A reductase (HMGCR). The subsequent depletion of cellular cholesterol activates proteolytic cleavage of membrane-bound sterol regulatory element-binding protein (SREBP)-2 in the endoplasmic reticulum (ER). The released N-terminal domain migrates into the nucleus and acts as a transcription factor to activate gene expression of the LDL receptor (LDLR) and other genes to rebalance cellular cholesterol homeostasis [3, 4]. Statins are overall safe with a remaining risk for raised transaminase levels and increased incidents of new onset diabetes (about 50–100 cases in 10,000 patients treated for 5 years), which is much outweighed by lowered CVD risk [5].

It is suspected that, in susceptible patients, statin-induced upregulation of LDLR results in cellular cholesterol overload, which triggers dysfunction of insulin-producing pancreatic β-cells. These cells are sensitive to excessive lipid import since they express only low levels of the antioxidants catalase, sodium dismutase (SOD)1 and SOD2 compared to other cell types, especially hepatocytes [6–8]. Their low antioxidant capacity makes β-cells susceptible to increased lipid oxidation and associated production of reactive oxygen species (ROS). Moreover, statin-mediated HMGCR inhibition leads to the depletion of isoprenoids like farnesyl pyrophosphate (FPP), which is a G protein-coupled receptor (GPR)92 agonist [9]. GPR92 is present in pancreatic islet macrophages and its activation with FFP reduced high-fat diet-induced islet inflammation and increased insulin secretion in wild type but not in GPR92 knockout (KO) mice [10].

In support of the assumed statin-mediated lipotoxicity, a number of in vitro studies observed that LDL-C exposure impairs insulin secretion and survival in rodent pancreatic islets and β-cell lines [11–14], which was not observed in pancreatic islets from *Ldlr* KO mice [12]. Similarly, increased cholesterol levels in islets from apolipoprotein E (*Apoe*) KO mice correlate with impaired glucose-stimulated insulin secretion, which is further worsened in obese *ob/ob;Apoe* KO mice [15]. The harmful effects of cholesterol accumulation are associated with activated signalling of c-Jun N-terminal kinase [14] and p38 mitogen-activated protein kinase as well as oxidative stress due to lipid oxidation [16].

In addition, mice with β-cell specific SREBP-2 overexpression develop severe diabetes due to loss of β-cell mass and function [17]. The idea of critical LDL-C overload in β-cells of statin-treated patients is further supported by the contradictory situation in patients with hypercholesterolemia due to mutations in *APOB* and *LDLR* genes [18]. These patients exhibit high plasma levels of LDL-C and a substantial risk for CVD, but display a lower prevalence of type 2 diabetes (T2D) than unaffected relatives.

Statins are meanwhile challenged by a new class of inhibitors that target the proprotein convertase subtilisin/kexin type 9 (PCSK9), the natural inhibitor of the LDLR [19]. The currently available PCSK9 inhibitors are the antibodies alirocumab and evolocumab. These antibodies bind to PCSK9, preventing its interaction with LDLR, which is then not targeted for degradation and becomes recycled repeatedly. Consequently, inhibition of PCSK9 increases numbers of LDLR on the cell surface. This results in LDL-C lowering by about 60%, a value that is comparable to high-intensity statin therapy (50–60%) [20]. Alirocumab and evolocumab are safe overall and so far without any indication for a diabetes risk [21, 22].

With regard to the common upregulation of LDLR, clinical PCSK9 inhibition is suspected to confer a similar diabetes risk as statins. Ongoing research has intensively investigated this point and widened the view on the multifaceted functions of PCSK9 beyond LDL-C lowering [19, 23]. In the following, we present the current understanding of the biology of PCSK9 and the consequences of loss-of-function mutations and clinical inhibition relevant to function and dysfunction of pancreatic β-cells.

## PCSK9 and cholesterol homeostasis

Cholesterol is an essential component of cell membranes and functional cholesterol homeostasis is of vital importance for all living cells including insulin producing pancreatic β-cells. However, cholesterol homeostasis must be carefully balanced. Cholesterol overload alters lipid raft composition and membrane fluidity, leading to a reduction of cell surface glucose transporters, increased glucokinase retention in insulin granules and altered Ca2 + and K + channels [24]. Its impact on mitochondrial membranes reduced energy production by the electron transport chain, increased ROS production and reduced antioxidants (glutathione), leading to mitochondrial stress and cellular apoptosis. In addition, cholesterol accumulation in ER membranes depletes Ca2 + storage, thus increasing ER stress.

Nevertheless, cells basically need cholesterol, which can be absorbed from food via the intestinal transporter Niemann-Pick C1-Like 1 (NPC1L1). Inhibition of NPC1L1 by ezetimibe lowered LDL-C levels by about 20% [20, 25]. Thus, most cholesterol comes from endogenous synthesis or storage. Although most cells are capable of synthesising cholesterol, the liver is of central importance as it contributes about 50% of circulating cholesterol and is also the main site for elimination of cholesterol from the bloodstream [26].

The liver releases acquired or newly synthesised cholesterol as very low-density lipoprotein (VLDL), which is processed in the blood stream to generate LDL for LDLR-mediated uptake into all cells of the organism [27]. LDL/LDLR complexes undergo endocytosis. LDLR dissociates from its ligand due to pH change during the late endosome to lysosome transition and becomes recycled. PCSK9 disrupts LDLR recycling through its binding to the extracellular epidermal growth factor (EGF)-A domain of LDLR, which is stabilized by acidic lysosomal pH, leading to the degradation of PCSK9/LDLR complexes [28]. A process that is also considered the main route to remove circulating PCSK9 from the bloodstream [29].

The liver is also central for the release of circulating PCSK9. Hepatocytes appear to be the only source, as circulating PCSK9 is not detectable in mice with liver-specific deletion [30]. Similar profiles of circulating LDL-C levels in mice with *Ldlr* KO and combined *Ldlr*/*Pcsk9* double KO indicate that PCSK9 regulates cholesterol homeostasis exclusively through LDLR. Hepatic expression and circulating levels of PCSK9 decrease during fasting and increase with food intake [31, 32]. PCSK9 is mainly regulated by SREBPs, in particular SREBP-2, which is also the principle activator of LDLR gene expression [33].

Newly translated pro-PCSK9 gets autocatalytically cleaved in the ER but remains associated to its pro-domain [19]. Both cleavage and pro-domain facilitate the exit of PCSK9 from the ER towards the Golgi for maturation and subsequent vesicle transport towards the cell surface for secretion. In vitro experiments suggest that PCSK9 can already bind LDLR intracellularly in order to direct it from the *trans*-Golgi network to lysosomal degradation [34]. However, the functional significance of the proposed internal degradation pathway remains unknown in vivo [19].

### Expression in extrahepatic tissues

Beyond the liver, PCSK9 is also expressed in many other tissues, including intestine, lung, kidney, the brain and pancreatic β-cells [30, 35]. In this regard, circulating cholesterol is reduced by 42% in mice with systemic *Pcsk9* KO and by 27% in mice with liver-specific KO [30]. Thus, about one third of PCSK9-mediated cholesterol regulation in mice is independent of circulating PCSK9, indicating a functional role of local PCSK9 in extrahepatic tissues.

This notion has been further evaluated in the heart. *Pcsk9* KO mice exhibit reduced running resistance associated with increased left ventricular wall thickness suggestive of heart failure with preserved ejection fraction (HFpEF) [36]. This was not observed in wild type controls and mice with liver-specific *Pcsk9* gene deletion. The development of HFpEF in *Pcsk9* KO mice was associated with impaired energy metabolism in cardiomyocytes and was not changed in *Ldlr/Pcsk9* double KO mice. Thus, PCSK9 mediates its effects on cardiomyocyte energy metabolism independent of LDLR by locally expressed PCSK9.

In this regard, PCSK9 exerts multiple functions beyond the regulation of LDLR [19]. Notably, PCSK9 targets the free fatty acid (FFA) scavenger CD36 and VLDL receptor (VLDLR) for degradation [37, 38]. Consequently, mice with *Pcsk9* KO show increased uptake of FFA and triglycerides leading to accumulation of lipid droplets in hepatocytes, hypertrophy of adipocytes and a substantially enlarged visceral adipose tissue. This is relevant to the overall situation in obesity, as in particular, the increase in visceral adipose tissue is closely associated with low-grade inflammation and insulin resistance in mice and patients [39]. A negative regulation of CD36 and VLDL by PCSK9 has also been shown in human pancreatic EndoC-βH1 β-cells [40]. PCSK9 is expressed in human pancreatic β-cells [41, 42], suggesting that combined limitation of cholesterol, FFA and triglyceride import compensates for the low antioxidant capacity of β-cells and their sensitivity to ROS generation during increased lipid oxidation [6, 7].

### Cholesterol export

**Fig. 1:**
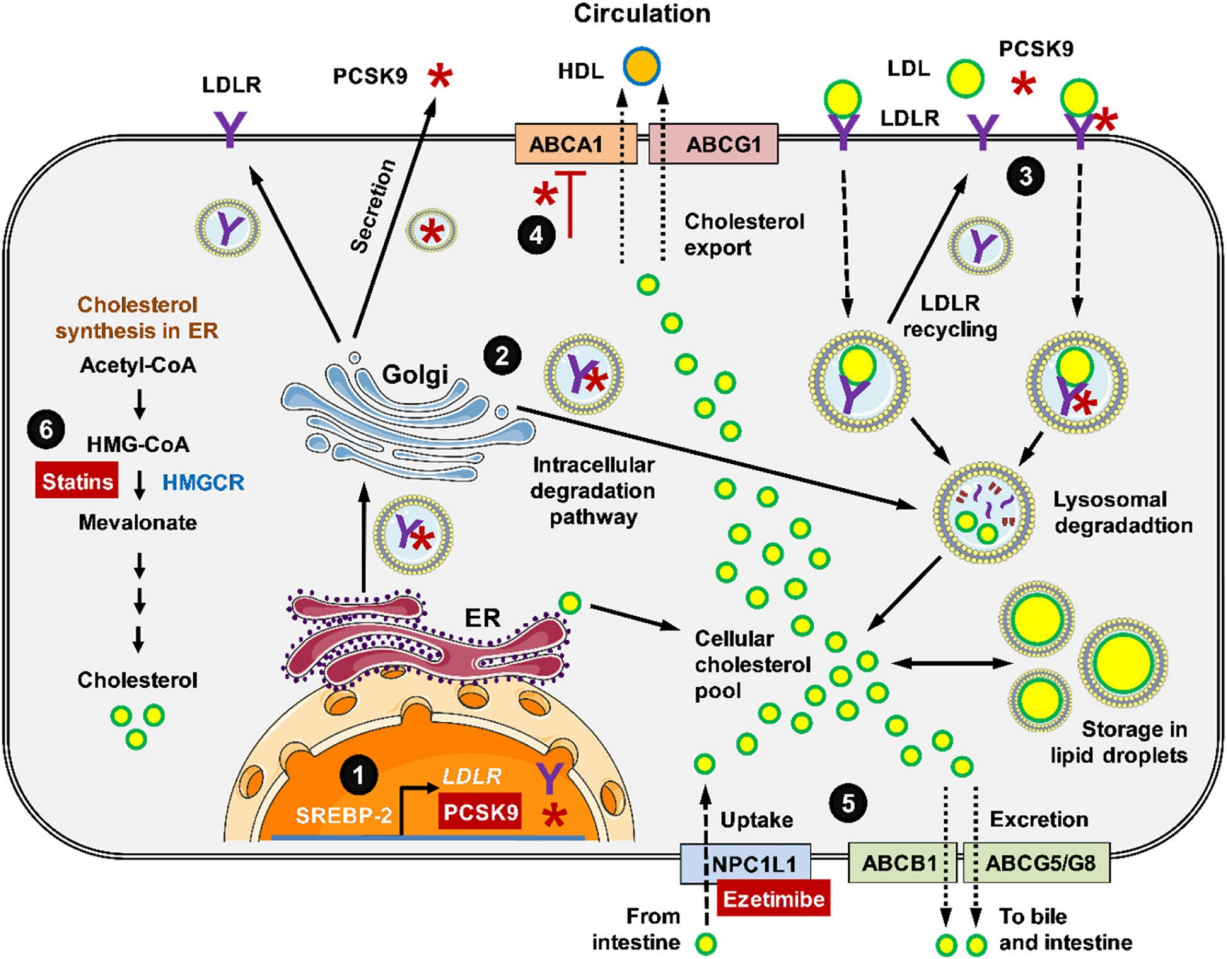
Schematic overview of cellular regulation of PCSK9 expression, processing, secretion, interaction with LDLR and degradation. (1) Gene expression of both PCSK9 and LDLR is induced by SREBP-2. (2) After maturation in the Golgi, PCSK9 is either routed to exocytosis or is intended together with LDLR to degradation in lysosomes. (3) LDL binds LDLR and both will be internalized. LDLR will be recycled if it is not bound to PCSK and thereby marked for degradation. (4) PCSK9 inhibits the expression of ABCA1, which together with ABCG1 exports intracellular surplus cholesterol to HDL formation in the blood stream and subsequent RCT to liver or TICE. (5) Cholesterol can also be excreted to bile or intestine via ABCG5/ABCG8 and ABCB1a/b. As alternative LDL-lowering drugs, (5) ezetimibe inhibits NPC1L1-mediated cholesterol uptake from intestine and (6) statins inhibit HMGCR-mediated cholesterol synthesis. The figure contains elements from Smart Servier Medical Arts (https://smart.servier.com/category/cellular-biology/intracellular-components/)

Cellular cholesterol homeostasis not only depends on regulated import but also on balanced export and high-density lipoprotein (HDL) generation (see Fig. 1 for overview). Mechanistically, surplus cellular cholesterol can be stored in lipid droplets or exported by the ATP-binding cassette (ABC)A1 and ABCG1 efflux transporters to apoA-I, which generates HDL-C destined for uptake into the liver via the scavenger receptor class B type 1 (SR-B1) [43]. A process referred to as reverse cholesterol transportation (RCT). Increased HDL-C blood levels have been associated with a lower risk of CVD, although the exact role of HDL in this context is still debated [44]. Beneficial effects of HDL have been attributed to its anti-inflammatory, antioxidant and antithrombotic functions [45]. These properties have been shown protective for β-cells. In vitro, HDL-incubation protects mouse β-TC-3 β-cells from LDL-induced apoptosis [12] and decreased basal and IL-1β-induced apoptosis in mouse and human islets [13]. HDL exposure further improved insulin secretion up to 5-fold in mouse MIN6 β-cells [46]. Consistently, T2D patients who received a 4-hour infusion of reconstituted HDL showed significantly increased insulin secretion and decreased blood glucose levels, with no change in insulin sensitivity [1].

The critical role of functional cholesterol export in insulin producing β-cells has been further shown in heterozygote Tangier patients with validated loss-of-function (LOF) gene variants of the *ABCA1* gene. These patients show a 60% reduction in HDL-C levels with normal LDL-C levels and insulin sensitivity, but substantially impaired first-phase insulin secretion during hyperglycaemic clamps [47]. Similarly, mice with β-cell-specific deletion of *Abca1* displayed impaired glucose tolerance [48], which worsened to substantial islet inflammation and diabetes in mice with additional deletion of *Abcg1* [49]. In this regard, patients treated with PCSK9 inhibitors showed no worsening of glycaemia and consistent increase in HDL-C serum levels by 4.6 to 8.9% depending on trial and therapeutic regime [50]. This supports the idea that ABCA1 and ABCG1 are capable to maintain critical cholesterol efflux in states of augmented LDL-C influx due to clinical PCSK9 inhibition. Consistent with this, PCSK9 has been shown to inhibit *Abca1* gene expression and export function in mouse macrophages [51]. Thus, inhibition of PCSK9 simultaneously increases both LDLR-mediated cholesterol import and its export via ABC transporters.

In addition to cellular storage and export towards HDL formation, excess cholesterol can also be eliminated from the organism by excretion to the bile or intestine. In both routes, cholesterol excretion is mediated by ABCG5/ABCG8 and ABCB1 transporters [52]. PCSK9 also appears to inhibit these cholesterol transporters since *Pcsk9* KO mice show increased transintestinal cholesterol excretion (TICE), which decreases after the injection of recombinant human PCSK9 [52].

## PCSK9 and glucose homeostasis

The hepatic expression of PCSK9 is influenced by insulin and glucagon. Insulin-stimulated *Pcsk9* gene expression involves activation of *Srebp-2* in rat hepatoma cells and of *Srebp-1* in primary rat hepatocytes [53]. Vice versa, glucagon-treatment reduced hepatic *Pcsk9* mRNA levels in mice [54]. In contrast, it has also been reported that loss of insulin signalling in mice with shRNA-mediated knockdown of insulin receptor signalling lead to increased hepatic PCSK9 expression in association with decreased signalling of the mammalian target of rapamycin complex 1 (mTORC1) and upregulation of hepatocyte nuclear factor 1a (HNF1a) [55]. Potentially, this upregulation is a regulatory feedback to reduced mTORC1 activity in certain conditions.

In the human context, circulating PCSK9 levels were found to be similar in healthy subjects and T2D patients [56] with no change in both groups upon 24 h exposure to moderate hyperinsulinemia [57]. In support, plasma PCSK9 levels in IT-DIAB (233 patients with prediabetes, follow-up 5 years) and the ELSA-Brasil (1,751 patients, 27.5% with prediabetes, follow-up 4 years) were not significantly associated with new-onset diabetes or glucose homeostasis, except for a positive correlation with insulin resistance in ELSA-Brasil [58]. These data do not favour an important link between circulating insulin and PCSK9. Likely, insulin and glucagon are rather fine-tuning hepatic PCSK9 expression than to be major regulators. Nevertheless, PCSK9 has been epidemiologically shown to mediate 11% of the association between depression and insulin resistance in a cohort of 389 obese patients [59].

Local PCSK9 expression in islets and β-cells has been assumed to compensate for the loss of circulating PCSK9 upon clinical inhibition or liver-specific gene deletion in mice. This is different in carriers of PCSK9 LOF variants since such mutations affect both local and circulating PCSK9. Several PCSK9 LOF variants have been reported with variable effects on glucose homeostasis [60]. For example, in a black South African population, variant A443T had no significant effect on glucose homeostasis, while C679X carriers showed reduced fasting glucose levels, albeit without changes in HbA1c [61, 62]. Also French Canadian carriers of the insLEU variant showed normal glycaemia [63]. However, individuals with additional familial hypercholesterolemia displayed a trend for higher incidence of prediabetes and diabetes as well as slightly but significantly increased plasma glucose levels [64]. Similarly, French and French Canadian carriers of the variant R46L also showed normal glucose homeostasis [63, 65] unless additional *APOE3/E2* polymorphism resulted in a trend towards insulin resistance with higher plasma insulin levels [63].

The meaning of these findings from small populations was further investigated at large-scale by a mendelian randomisation study using data from 550,000 individuals including 51,623 cases of T2D [66]. The combined analyses of four LDL lowering PCSK9 gene variants finds an association with elevated fasting glucose levels and an increased risk of T2D [odds ratio of 1.29 (1.11 to 1.50)]. The individual risk of the variants was depending on the individual extent of functional loss, with the highest risk of diabetes occurring in the variant with the highest LDL-C lowering (lowest PCSK9 function). However, a complete functional loss of PCSK9 is only well defined in animal knockout models.

An initial study generated *Pcsk9* KO mice by inbreeding *Pcsk9+/-* mice from The Jackson Laboratories (Maine, USA) and reported strong upregulation of LDLR on β-cells, but normal islet cholesterol content and functional insulin secretion [67]. Another study backcrossed the Jackson Lab *Pcsk9+/-* males with B6 females before generating the *Pcsk9* KO. These mice showed accumulation of cholesterol in islets and impaired glucose-stimulated insulin secretion irrespective of gender and diet [68, 69]. However, only males but not females showed substantial worsening of glucose tolerance, which was further similar between wild type and KO. Probably, KO genotype was superimposed by the genetic predisposition to insulin resistance and diabetes in aged male mice. Note that healthy mice were studied at 2–3 months of age and mice with impaired insulin secretion at 4–6 months of age.

The initial findings have been readdressed by comparing an alternative systemic KO and liver-specific gene deletion [70]. Systemic loss in these *Pcsk9* KO mice aged 5–7 months (no gender indicated) was associated with increased cholesterol accumulation in islets and impaired insulin secretion independent of normal diet or 20 weeks high fat diet. The disturbed insulin exocytosis goes along with increased insulin storage and results in significant but moderate loss of glucose tolerance. In contrast, mice with liver-specific *Pcsk9* KO remained normoglycaemic. The same applies to 3 months old (male) mice with β-cell-specific *Pcsk9* KO [71]. However, in this study insulin response and sensitivity were also not worsened in 4.5 months old mice with systemic *Pcsk9* KO. Therefore, the phenotype of moderate β-cell dysfunction is not consistent in different models of systemic *Pcsk9* KO, but so far absent in mouse models with liver- and β-cell-specific KO.

All in all, this supports the idea that PCSK9 tissue expression in islets compensates for loss of liver-derived PCSK9 in circulation. PCSK9 is expressed in the rodent β-cell lines RIN-m5F, β-TC-3 [35], MIN6 and in mouse islets [69] as well as in human EndoC-βH1 β-cells [42] and human islets [41]. β-cells appear to be an abundant, but not the only source for PCSK9 in endocrine pancreas since islets from mice with β-cell-specific *Pcsk9* KO showed substantial but not complete reduction of PCSK9 mRNA and protein by 48% and 78%, respectively [71]. Similarly, sorted human islet cells from healthy donors showed marked *Pcsk9* gene expression in β-cell enriched fractions, which was less pronounced in fractions enriched of other islet cell types [41].

These findings point out that PCSK9 is still expressed in islets after β-cell-specific loss, e.g. by δ-cells [67, 70, 72]. This plausibly explains maintained β-cell function in mice with β-cell-specific *Pcsk9* KO [71]. The situation changed in mice with pancreas-specific *Pcsk9* KO and complete loss of all local PCSK9 in islets, which showed a moderately impaired insulin response and glucose tolerance [72]. However, the situation is contradictory in vitro. Insulin response was not significantly altered in human EndoC-bH1 β-cells treated with recombinant PCSK9, alirocumab or siRNA-mediated PCSK9 knockdown [42]. In contrast, siRNA-mediated silencing of PCSK9 in rat INS-1E β-cells significantly reduced glucose-induced insulin response in association with decreased levels of vesicle-related soluble N-ethylmaleimide-sensitive factor attachment protein receptor (SNARE) proteins [72]. If not cell line-specific, this would suggest a role for PCSK9 in the secretory pathway.

## Bottom down

**Fig. 2:**
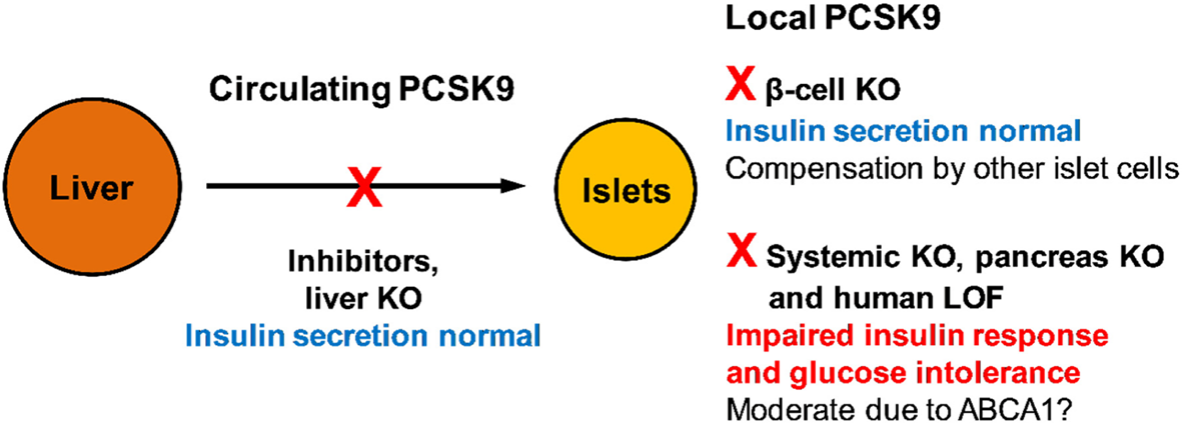
Summary of the effects of different types of PCSK9 loss on glucose homeostasis. Mice with loss of liver-derived circulating PCSK9 or partial loss of local expression in islets upon β-cell-specific KO remain normoglycaemic. Systemic *Pcsk9* KO (not in all models) and loss of all local PCSK9 throughout pancreas resulted in moderately reduced insulin response and glucose tolerance. This underlines the importance of local PCSK9 expression in pancreatic islets for proper β-cell function. Similarly in patients, inhibition of circulating PCSK9 by alirocumab and evolocumab has no effect on glucose homeostasis, whereas loss of local tissue-derived PCSK9 in patients with LOF variants leads to worsening of glycaemia depending on the degree of functional loss. In general, the deterioration of glucose homeostasis observed under certain conditions was moderate. Possibly, the assumed lipotoxicity of increased LDLR-mediated cholesterol import was counter-regulated by increased cholesterol export via ABCA1. KO, knockout; inhibitors, alirocumab and evolocumab; LOF, loss-of-function

The cholesterol homeostasis in insulin producing β-cells appears to be robust to loss of circulating PCSK9, as mice with liver-specific *Pcsk9* gene deletion and inhibitor-treated patients show no significant worsening of insulin secretion or glycaemia (see Fig. 2 for summary). PCSK9 is locally expressed in islets by δ-cells and β-cells. Since PCSK9 targets not only LDLR but also VLDLR and CD36, it can be assumed that its local expression in islets protects β-cells from lipid overload. Note that β-cells have only a very low antioxidant capacity and are therefore sensitive to generation of ROS during lipid oxidation. Local PCSK9 expression in islets appears to be essential for β-cell-function since its loss in mice with systemic and pancreas-specific *Pcsk9* KO resulted in moderate impairment of insulin secretion and glucose tolerance. However, β-cell dysfunction is not present in all mouse models with systemic *Pcsk9* KO. Impairment of glycaemia is also variable in human carriers of PCSK9 LOF variants, where the diabetes risk correlates with the individual degree of functional loss. The overall rather moderate deterioration of glucose homeostasis and diabetes risk could indicate a compensatory cholesterol export via ABCA1 and ABCG1. In macrophages, ABCA1 is inhibited by PCSK9 and thus activated by its functional loss. Collectively, the current molecular and metabolic evidence supports the notion of the safety of PCSK9 inhibitor therapy. However, prolonged use, future clinical trials and meta-analyses may define yet undiscovered patients at risk for diabetes.

## Data Availability

Not applicable.

## Abbreviations

Apo: Apolipoprotein
ABC: ATP binding cassette
CVD: Cardiovascular disease
CD36: Cluster of differentiation 36
ER: Endoplasmic reticulum
FPP: Farnesyl pyrophosphate
GPR: G protein-coupled receptor
HNF1a: Hepatocyte nuclear factor 1a
HDL: High-density lipoprotein cholesterol
HMGCR: 3-hydroxy-3-methyl-glutaryl-coenzyme A reductase
LOF: Loss-of-function
LDL: Low-density lipoprotein
LDLR: Low-density lipoprotein receptor
mTORC1: Mammalian target of rapamycin complex 1
NPC1L1: Niemann-Pick C1-like 1
PCSK9: Proprotein convertase subtilisin/kexin type 9
RCT: Reverse cholesterol transportation
SNARE: Soluble N-ethylmaleimide-sensitive factor attachment protein receptor
SOD: Sodium dismutase
SR-B1: Scavenger receptor class B type 1
SREBP: Sterol regulatory element-binding protein
TICE: Transintestinal cholesterol excretion
T2D: Type 2 diabetes
VLDL: Very low-density lipoprotein
